# Effects of age and sex on cerebrovascular function in the rat middle cerebral artery

**DOI:** 10.1186/s13293-014-0012-8

**Published:** 2014-09-11

**Authors:** Rachel R Deer, John N Stallone

**Affiliations:** 1 Women’s Health Division, Michael E. DeBakey Institute, Texas A & M University, College Station 77843-4466, TX, USA; 2 Department of Veterinary Physiology and Pharmacology, College of Veterinary Medicine and Biomedical Sciences, Texas A & M University, College Station 77843-4466, TX, USA; 3 Sealy Center on Aging, University of Texas Medical Branch, 301 University Boulevard, Galveston 77550-0177, TX, USA

**Keywords:** Cerebrovascular, Sex-differences, Vasoconstriction, Sexual dimorphism, Cyclooxygenase, Thromboxane

## Abstract

**Background:**

Although the mechanisms underlying the beneficial effects of estrogen on cerebrovascular function are well known, the age-dependent deleterious effects of estrogen are largely unstudied. It was hypothesized that age and sex interact in modulating cerebrovascular reactivity to vasopressin (VP) by altering the role of prostanoids in vascular function.

**Methods:**

Female (F) Sprague–Dawley rats approximating key stages of “hormonal aging” in humans were studied: premenopausal (mature multigravid, MA, cyclic, 5–6 months) and postmenopausal (reproductively senescent, RS, acyclic, 10–12 months). Age-matched male (M) rats were also studied. Reactivity to VP (10^−12^–10^−7^ M) was measured in pressurized middle cerebral artery segments in the absence or presence of selective inhibitors of COX-1 (SC560, SC, 1 μM) or COX-2 (NS398, NS, 10 μM). VP-stimulated release of PGI_2_ and TXA_2_ were measured using radioimmunoassay of 6-keto-PGF_1α_ and TXB_2_ (stable metabolites, pg/mg dry wt/45 min).

**Results:**

In M, there were no changes in VP-induced vasoconstriction with age. Further, there were no significant differences in basal or in low- or high-VP-stimulated PGI_2_ or TXA_2_ production in younger or older M. In contrast, there were marked differences in cerebrovascular reactivity and prostanoid release with advancing age in F. Older RS F exhibited reduced maximal constrictor responses to VP, which can be attributed to enhanced COX-1 derived dilator prostanoids. VP-induced vasoconstriction in younger MA F utilized both COX-1 and COX-2 derived constrictor prostanoids. Further, VP-stimulated PGI_2_ and TXA_2_ production was enhanced by endogenous estrogen and decreased with advancing age in F, but not in M rats.

**Conclusions:**

This is the first study to examine the effects of age and sex on the mechanisms underlying cerebrovascular reactivity to VP. Interestingly, VP-mediated constriction was reduced by age in F, but was unchanged in M rats. Additionally, it was observed that selective blockade of COX-1 or COX-2 produced age-dependent changes in cerebrovascular reactivity to VP and that VP-stimulated PGI_2_ and TXA_2_ production were enhanced by endogenous estrogen in younger F. A better understanding of the mechanisms by which estrogen exerts its effects may lead to new age- and sex-specific therapeutic agents for the prevention and/or treatment of cerebrovascular diseases.

## 1 Background

The human aging process is associated with marked sexual dimorphism in the incidences of neurological and vascular diseases, but the reasons for these sex differences in disease are unclear [1]. Premenopausal women exhibit lower incidences of cardiovascular disease and stroke than males of the same age, yet after menopause, these differences dissipate. Because the risks of cardiovascular disease and stroke increase with the onset of menopause, estrogen has been implicated as protective against these diseases. Indeed, estrogen appears to play a fundamental role in the maintenance of both neuronal and vascular health in younger women and to be protective against diseases such as coronary artery disease, hypertension, and stroke [2]–[5]. In numerous animal studies, estrogen exerts beneficial effects by (1) enhancing vasodilator factors, including nitric oxide and prostacyclin (PGI_2_), (2) increasing expression and/or activity of endothelial nitric oxide synthase (eNOS), cyclooxygenase-1 (COX-1), and prostacyclin synthase proteins, and (3) stimulating the phosphatidylinositol 3-kinase/Akt pathway, which increases eNOS phosphorylation, eNOS activity, and subsequently nitric oxide production [6].

Disruption of the endocrine environment, both during menopause and with advancing age, contributes to dramatic increases in the incidence of neurodegenerative and vascular diseases, especially stroke. While both endogenous estrogens and estrogen replacement therapy following surgical menopause exert beneficial effects in younger females (F), age and/or estrogen replacement therapy appear to be detrimental in older, postmenopausal F. In fact, epidemiological and experimental studies revealed that both age and estrogen replacement therapy increase the risk for stroke and the extent of brain injury following ischemic stroke in aged F [7]. Recent studies in the systemic vasculature demonstrated that estrogen enhances the production of, and reactivity to, thromboxane (TXA_2_) and other deleterious constrictor prostanoids in the F Sprague–Dawley rat [8],[9]. Thus, it is important to determine how age and sex interact in the regulation of cerebrovascular prostanoid production and the enhancement of both vasoconstriction and hemostasis, and how these mechanisms may differ between males (M) and F with age.

It is difficult to reconcile the apparent conflict in beneficial versus deleterious effects of estrogen on neurological and vascular function unless the effects of age are considered. Although the mechanisms underlying the beneficial effects of estrogen on cerebrovascular function have been studied extensively, studies on the mechanisms responsible for deleterious effects of age and sex on the modulation of cerebrovascular reactivity are extremely limited. This lack of understanding emphasizes the importance of examining the cellular and molecular mechanisms underlying the deleterious effects of age, sex, and endogenous estrogen on the cerebral vasculature. Thus, in the present studies, the central hypothesis tested is that age and sex interact in modulating cerebrovascular reactivity to vasopressin (VP) by altering the role of prostanoids in vascular function.

## 2 Methods

### 2.1 Ethical approval

All animal protocols were in accordance with “U.S. Government Principles for the Utilization and Care of Vertebrate Animals Used in Testing, Research and Training” as detailed in the National Institutes of Health “Guide for the Care and Use of Laboratory Animals” and approved by the Texas A & M University Institutional Animal Care and Use Committee.

### 2.2 Animals and maintenance

Neurological and vascular effects of estrogen, in both humans and experimental animals, appear to depend upon age. Thus, female (F) Sprague–Dawley rats of differing age groups, which approximate two key stages of “hormonal age” in humans, were studied. Mature, multigravid, F rats (MA, 5–6 months age) are older than the virgin F used in most studies, have carried 2–3 previous pregnancies, and exhibit a longer and somewhat irregular 5–9-day estrous cycle. Reproductively senescent (RS, 12–14 months age) are the oldest F, have carried 5–6 previous pregnancies, and are acyclic. These two different groups of F rats are intended to model two distinctly different hormonal age groups of women, namely, mature (perimenopausal) and aged (postmenopausal). This model has been used successfully to study the effects of age and estrogen on neurological function and excitotoxic injury [10]–[13]. Age-matched male (M) rats were also studied as a control for the effects of aging. For the independent effects of age, it was important to identify the phases of the estrous cycle in female experimental animals, and thus, daily vaginal smears were used to determine the stage of the estrous cycle at the time of experimentation. To eliminate the potential effects of cyclic surges in estrogen on vascular function, F rats were only used for experiments while in metestrus/diestrus phases, although, previous studies failed to detect changes in systemic vascular reactivity to VP or phenylephrine [14]. All rats were purchased from Harlan (Houston, TX, USA) and housed at the main animal facility at Texas A & M University. Rats were housed in pairs, in standard plastic laboratory rat cages, in a well-ventilated room, maintained at constant temperature (21°C–26°C), and controlled photoperiod (12 h light: 12 h dark). Sixteen percent protein global diet (soy and alfalfa-free to minimize dietary phytoestrogens, Harlan Teklad diets, Houston, TX, USA) and water were provided *ad libitum*.

### 2.3 Plasma estrogen concentration

F rats typically have a short estrous cycle lasting 4–5 days. Plasma estrogen concentrations oscillate markedly during the phases of the cycle, attaining a peak surge during proestrus and a nadir during metestrus. Because the proposed studies focus on the effects of estrogen levels and age, the endocrine status of all F rats was determined by vaginal smear. Smears were taken daily, over 2–3 consecutive cycles (or for approximately 21 days), immediately preceding, and including, the day of animal sacrifice and blood collection for measurement of estrogen. Estrous phase was determined by histological characteristics of the smears [15],[16]. Trunk blood was collected from all animals in ethylenediaminetetraacetic acid (EDTA)-coated tubes at the time of sacrifice, centrifuged, and the plasma was stored at −80°C for later analysis of plasma 17β-estradiol levels by radioimmunoassay (RIA).

### 2.4 Pressurized cannulated MCA vessel preparation

Age-matched M and F rats (mature, MA and reproductively senescent, RS) were humanely euthanized by rapid decapitation to avoid artifactual effects of anesthetics and minimize activation of neural and humoral pathways. The middle cerebral arteries (MCA) were isolated immediately and placed in chilled Krebs-Henseleit bicarbonate solution (KHB). The KHB was composed of (in mM) 118.0 NaCl, 25.0 NaHCO_3_, 10.0 glucose, 4.74 KCl, 2.5 CaCl_2_, 1.18 MgSO_4_, and 1.18 KH_2_PO_4_. MCAs from each animal were cleaned of connective and brain tissue, and arterial segments were prepared in triplicate. They were cannulated and tied securely to the pipettes using 11–0 ophthalmic suture. The glass micropipettes were filled with physiological salt solution (PSS) with albumin, which contained the following (in mM): 145 NaCl, 4.7 KCl, 2.0 CaCl_2_, 1.17 MgSO_4_, 3.0 MOPS, 1.2 NaH_2_PO_4_, 5.0 glucose, 2.0 pyruvate, 0.02 EDTA, and 1% bovine serum albumin (BSA). The cannulated vessel was transferred to the stage of an inverted microscope (Olympus CKX41, Olympus, Shinjuku, Japan) equipped with a × 4 objective (numerical aperture of 0.13) and coupled with a video camera (Hitachi KP-M3AN, Hitachi, Chiyoda-ku, Japan), video monitor (Pelco PMM12A, Pelco, Clovis, CA, USA), DVD recorder (Phillips DVDR3475, Phillips, Amsterdam, The Netherlands), and video micrometer (Colorado Video 307A, Boulder, CO, USA). Both micropipettes were connected to a single reservoir system and were gradually adjusted to set the intraluminal pressure of the vessel at 85 mmHg without allowing flow through the vessel lumen. Leaks were detected by verifying that intraluminal diameter of the pressurized arteriole remained constant when the valve to the reservoir system was closed. Only arterioles that were free of leaks were studied. The vessel chamber bath (Living Systems TC-09S, Living Systems, Miami, FL, USA) containing PSS + albumin was gradually warmed and maintained at 37°C for the duration of the experiment. Luminal diameter was monitored continuously throughout the experiment. The vessels were allowed to equilibrate for 1 h before being pretreated with pharmacological agents indicated below for 20 min. Cumulative concentration-response curves to arginine VP (10^−12^–10^−7^ mol/L) were obtained by direct, cumulative additions of VP into the tissue baths, in the absence or presence of inhibitors including (1) selective COX-1 inhibitor (SC560, 1 μM) or (2) selective cyclooxygenase-2 (COX-2) inhibitor (NS398, 10 μM). Diameter measurements were determined in response to cumulative concentrations of VP. Percent constriction was determined by the following equation: %constriction = (*B*_D_ − *B*_x_)/*B*_D_ × 100, where *B*_D_ is the steady-state baseline diameter after inhibitor incubation and *B*_X_ is the diameter after each VP concentration. The concentration of VP that produced 50% of the maximal response (EC_50_) was calculated individually from the log concentration-response curve of each MCA segment.

### 2.5 Prostanoid release assay (TXA_2_ and PGI_2_)

Vascular prostanoid production by MCA obtained from age-matched M and F rats (mature, MA and reproductively senescent, RS) was measured using incubation and radioimmunoassay methods adapted for microvessels, as described previously [17]. Briefly, isolated MCA (3–4 mm axial length) were cleaned of connective tissue and fat, placed into chilled PSS without BSA (PSS-BSA) to rest for 60 min. The arterial segments were then transferred into 0.5 mL microcentrifuge tubes with 450 μL chilled solution and gradually warmed in a water bath to 37°C for a 45-min pre-incubation. The pre-incubation medium was carefully aspirated and 300 μL PSS-BSA alone (basal) or PSS-BSA with VP 10^−9^ M (low) or PSS-BSA with VP 10^−7^ M (high) was added and incubated at 37°C for 45 min. After incubation, the incubation media were collected and stored at −80°C until RIA of stable metabolites of PGI_2_ (6-keto-prostaglandin F_1α_; 6-keto-PGF_1α_) and TXA_2_ (TXB_2_). MCA segments were saved and stored at −80°C for dry weight analysis.

### 2.6 Chemical reagents and drugs

The following reagents and drugs were used: 17β-estradiol (Innovative Research of America, Sarasota, FL, USA), SC560 and NS398 (Cayman Chemical, Ann Arbor, MI, USA), arginine VP (Bachem, Torrance, CA, USA). All other chemicals were of reagent grade quality and were purchased from Sigma Chemical (St. Louis, MO, USA).

### 2.7 Statistics

All data are expressed as means ± SE; *n* indicates the number of animals studied. One- or two-way analysis of variance (ANOVAs) was used to detect significant differences among means of all experimental groups. If a main effect was identified, pairwise Student's *t* tests were performed to detect significant differences between any two means of the data groups, with a Bonferroni correction for multiple comparisons. Vascular function and prostanoid release data were analyzed using a two-way ANOVA for sex (M vs. F) and age (MA vs. RS). The effects of treatment (control (CTL), COX-1 inhibition, COX-2 inhibition) were analyzed in each experimental group using a one-way ANOVA. Bonferroni multiple comparison correction was used. Plasma estradiol levels, body weight, and uterine weight were analyzed by sex and age using a two-way ANOVA and Student's *t* tests. A *P* value ≤ 0.05 was considered significant.

## 3 Results

### 3.1 Effects of age and sex on estrogen levels, body weight, and uterine weight

Due to the design of the experiments, the younger MA F were in metestrus or diestrus phase of the estrous cycle (as determined by vaginal smears) and thus were sacrificed during low, non-surge, estradiol levels. Nevertheless, plasma estrogen levels in MA F rats (11.25 ± 1.72 pg/mL) were nearly double those of acyclic RS F rats (6.59 ± 1.32 pg/mL; *P* < 0.023). In previous studies, randomly sampled plasma estradiol levels of young, intact, cycling females averaged 43.9 ± 13 pg/mL [9]. In contrast, plasma estrogen levels in M rats in the present study were substantially lower and nearly undetectable (MA M 2.54 ± 1.06 pg/mL; RS M 0.33 ± 0.09 pg/mL). Uterine weights did not significantly differ with age (MA F 0.41 ± 0.04 g/100 g body weight; RS F 0.34 ± 0.03 g/100 g body weight; *P* > 0.05). Body weight did not differ with age in F (MA F 303 ± 5.8 g; RS F 303 ± 9.5 g; *P* > 0.05); however, RS M were significantly heavier than MA M (MA M 491 ± 10.9 g; RS M 559 ± 12.3 g; *P* ≤ 0.01). Body weights were significantly different between M and F in both young MA and older RS rats (*P* ≤ 0.01).

### 3.2 Effects of age and sex on vascular reactivity to VP

**Figure 1:**
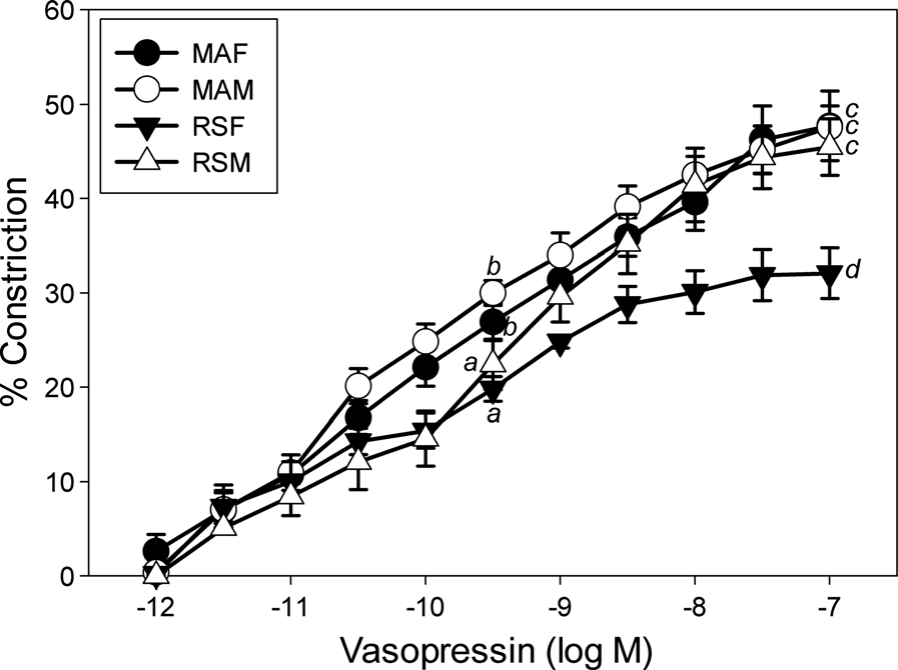
**VP concentration-response curves in endothelium-intact pressurized MCA segments.** Mature multigravid adult (MA, 4–6 months) female (MA F) or age-matched male rats (MA M) and reproductively senescent rats (RS, 10–12 months) female (RS F) or age-matched male rats (RS M). Data points represent means ± SE (*n* = 6 rats/group). (a-d) 0.0006 ≤ *P* ≤ 0.04, mean values without common superscript differ significantly at middle and maximal concentrations of VP.

**Figure 2:**
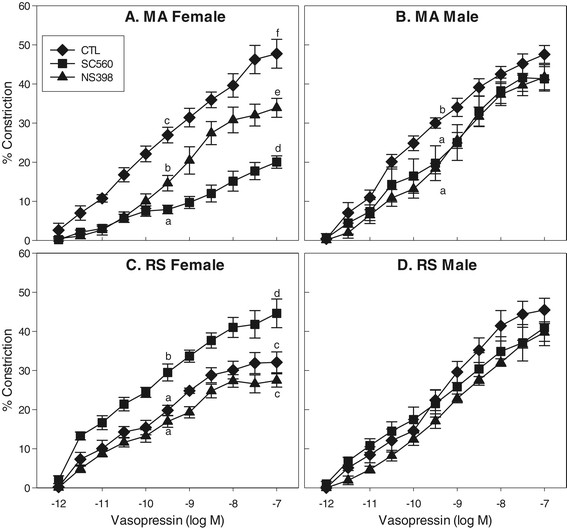
**VP concentration-response curves in MCA segments in the absence or presence of selective COX inhibitors.** Preparations were studied in triplicate from each animal group: mature multigravid adult (MA, 4–6 months) female (MA F) **(A)** or age-matched male rats (MA M) **(B)**, reproductively senescent (RS, 10–12 months) female (RS F) **(C)**, or age-matched male rats (RS M) **(D)**. Data points represent means ± SE (*n* = 6 rats/group). (a-f) 0.0001 ≤ *P* ≤ 0.02, mean values without common superscript differ significantly at middle and maximal concentrations of VP. In RS M, there were no significant differences among CTL, SC560, and NS398 groups.

The effects of age and sex on VP-induced vasoconstriction of MCA are shown in Figures 1 and 2 and Table 1. Comparison of control curves (Figure 1) revealed significant age differences in both M and F at the middle VP concentration. At the maximal VP concentration, age had a significant effect in F, but not in males, and in older RS rats, sex also had a significant effect. In older RS F rats, VP-stimulated constriction was attenuated significantly at both middle and maximal VP concentrations as compared with younger MA F. In both MA and RS M rats, VP-induced constriction did not differ significantly from MA F. At the middle VP concentration, constrictions to VP in RS M were significantly attenuated compared to MA F and MA M; however, sensitivity to VP did not differ.

**Table 1:** The effects of age and sex on VP-induced vasoconstriction of endothelium-intact pressurized MCA segments

**Group**	**Treatment**	**Maximal response to VP (% Constriction)**	**Relative maximal response to VP**	**Dilator PG**	**Constrictor PG**
MA F	Control	47.7 ± 3.7	↑	-	-
	COX-1	20.0 ± 1.6		0	↑↑↑
	COX-2	33.9 ± 2.4		0	↑↑
MA M	Control	47.6 ± 2.3	↑	-	-
	COX-1	41.3 ± 3.1		0	↑
	COX-2	41.7 ± 3.2		0	↑
RS F	Control	32.1 ± 2.7	↓	-	-
	COX-1	44.6 ± 3.7		↑↑	0
	COX-2	27.5 ± .7		0	0
RS M	Control	45.5 ± 3.0	↑	-	-
	COX-1	41.0 ± 4.6		0	0
	COX-2	39.7 ± 2.3		0	0

The effects of age and sex on VP-induced vasoconstriction of endothelium-intact pressurized MCA segments and the relative contributions of constrictor vs. dilator prostanoids in mature multigravid adult (MA, 4–6 months) female (F) (MA F) or age-matched male (M) rats (MA M) and reproductively senescent (RS, 10–12 months) female (F) (RS F) or age-matched male (M) rats (RS M). Vasopressin (VP). Prostaglandin (PG). Treatment reflects either control vascular reactivity or reactivity in the presence of COX-1 (SC560) or COX-2 (NS398) inhibition. Arrows indicate relative differences in response.

In MA F, VP produced concentration-dependent constrictions with a maximal response of 47.7% and an EC_50_ of 0.39 nM (Figure 2A). Both COX-1 and COX-2 selective inhibitors, SC560 and NS398, significantly attenuated constriction at middle and maximal VP concentrations. Compared with the control group, maximal constriction was reduced by 58% and 29% by SC560 and NS398, respectively.

Maximal constrictor response to VP was 32.1% in RS F with an EC_50_ of 0.13 nM (Figure 2C). Sensitivity to VP (EC_50_) did not differ with age in MA F vs. RS F. SC560 significantly enhanced maximal constriction to VP in RS F by 39%, yet NS398 had no significant effect. It is important to note that the reduction in VP constriction in RS F (as compared to MA F, MA M and RS M) was due to an enhancement in COX-1 derived dilator prostanoids.

In MA M, VP produced concentration-dependent constrictions with a maximal response of 47.6% and an EC_50_ of 0.10 nM (Figure 2B). At the middle VP concentration, both SC560 and NS398 attenuated constriction in MA M; however, these differences dissipated at the maximal VP concentration.

In RS M, VP produced concentration-dependent constrictions with a maximal response of 45.5% and an EC_50_ of 0.50 nM (Figure 2D). SC560 and NS398 had no significant effect on VP constrictions in older RS M rats at either middle or maximal VP concentrations.

### 3.3 Effects of age and sex on basal and VP-stimulated PGI_2_ release

**Figure 3:**
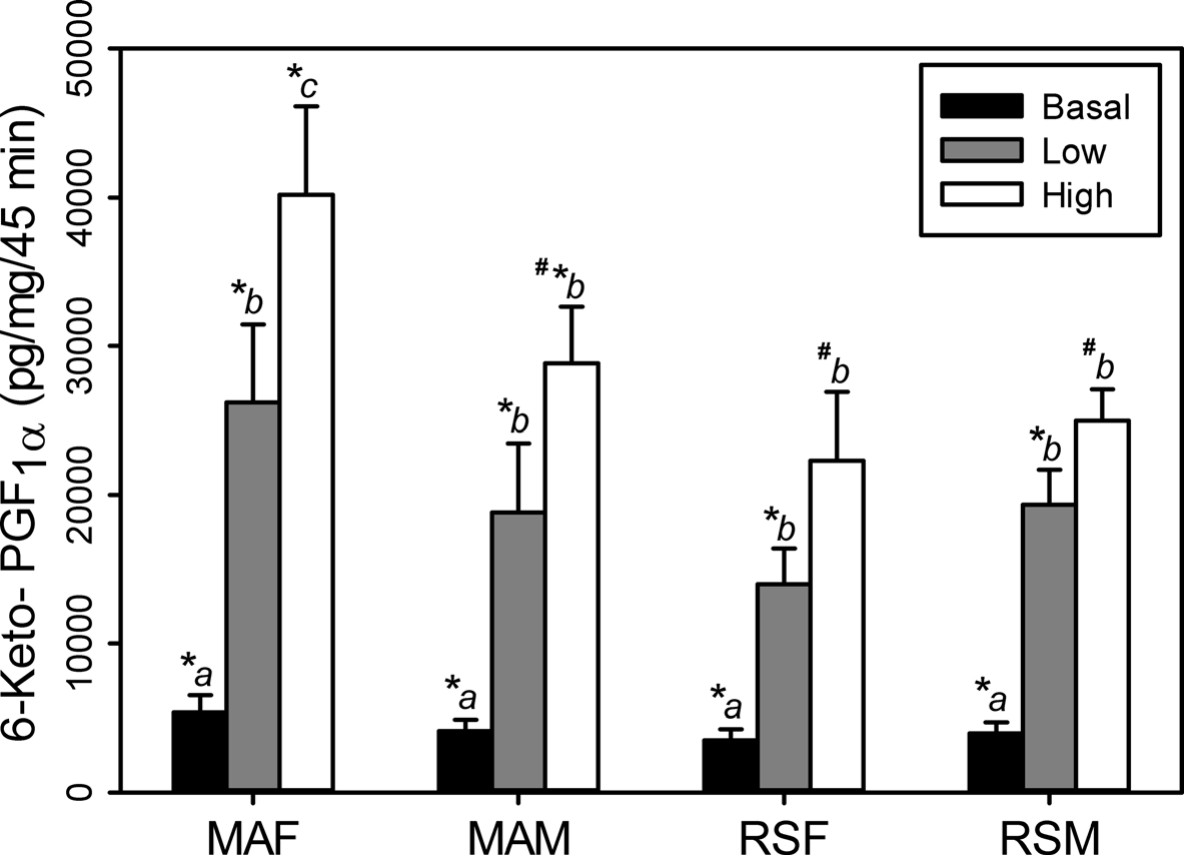
**Basal and VP-stimulated release of 6-keto-PGF**_**1α**_**by MCA segments.** Mature multigravid adult (MA, 4–6 months) female (MA F) or age-matched male rats (MA M) and reproductively senescent (RS, 10–12 months) female (RS F) or age-matched male rats (RS M). Data points represent means ± SE (*n* = 6 rats/group). (a-c) *P* ≤ 0.0001, mean values within groups (MA F, MA M, RS F, RS M) without common superscript are significantly different. (*, #) 0.0001 ≤ *P* ≤ 0.02 mean values between groups (MA F vs. MA M vs. RS F vs. RS M) with different superscripts are significantly different.

Basal and VP-stimulated (low concentration 10^−9^ M; high concentration 10^−7^ M) release of 6-keto-PGF_1α_ are shown in Figure 3. Basal and low concentration VP-stimulated release of 6-keto-PGF_1α_ did not differ significantly between groups (MA F, MA M, RS F, or RS M) (*P* > 0.05). Within each of the four groups, VP increased 6-keto-PGF_1α_ production in a concentration-dependent manner. Low-concentration VP-stimulated 6-keto-PGF_1α_ release did not differ significantly between groups. However, low-concentration VP increased 6-keto PGF_1α_ by fourfold in RS F and fivefold in MA F, MA M, and RS M, from their respective basal levels. High-concentration VP increased 6-keto-PGF_1α_ production eightfold in MA F, sevenfold in MA M, and sixfold in RS F and RS M from their respective basal levels. At high-concentration VP, MA F produced significantly more 6-keto PGF_1α_ than RS F or all other groups.

### 3.4 Effects of age and sex on basal and VP-stimulated TXA_2_ release

**Figure 4:**
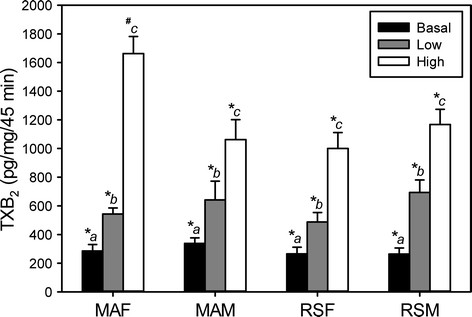
**Basal and VP-stimulated release of TXB**_**2**_**by MCA segments.** Mature multigravid adult (MA, 4–6 months) female (MA F) or age-matched male rats (MA M) and reproductively senescent (RS, 10–12 months) female (RS F) or age-matched male rats (RS M). Data points represent means ± SE (*n* = 6 rats/group). (a-c) 0.0001 ≤ *P* ≤ 0.003, mean values within groups (MA F, MA M, RS F, RS M) without common superscript are significantly different. (*, #) 0.0001 ≤ *P* ≤ 0.003 mean values between groups (MA F vs. MA M vs. RS F vs. RS M) with different superscripts are significantly different.

Basal and vasopressin-stimulated (low concentration 10^−9^ M; or high concentration 10^−7^ M) release of TXB_2_ are shown in Figure 4. Basal and low concentration VP-stimulated release of TXB_2_ did not significantly differ between groups (MA F, MA M, RS F, or RS M). Within each of the four groups, VP increased TXB_2_ production in a concentration-dependent manner. Low-concentration VP increased TXB_2_ production twofold in MA F, RS F, and MA M and threefold in RS M from their respective basal levels. High-concentration VP increased TXB_2_ production sixfold in MA F, threefold in MA M, and fourfold in RS F and RS M from their respective basal levels. At high-concentration VP, MA F produced significantly more TXB_2_ than all other groups.

## 4 Discussion

The present study is the first to examine the effects of age and sex on the mechanisms underlying cerebrovascular reactivity to VP in the MCA of Sprague–Dawley rats. The results reveal the new and novel findings that both age and sex alter cerebrovascular reactivity by modulating prostanoid production in the MCA. The major findings of this study are that (1) VP-induced constriction is reduced by age in F but is unchanged in M rats, (2) selective blockade of COX-1 or COX-2 produced greater age-dependent changes in cerebrovascular reactivity to VP in F than in M rats, and (3) VP-stimulated PGI_2_ and TXA_2_ production are reduced by age in F but not in M rats. In M, there were no changes in VP-induced vasoconstriction with age. Additionally, there were no significant differences in basal, low- or high-VP-stimulated PGI_2_ or TXA_2_ production as measured by the stable metabolites 6-keto-PGF_1α_ or TXB_2_ of young MA M or older RS M rats. In contrast, there were marked differences in cerebrovascular reactivity and prostanoid release with advancing age in F. Older F rats in this study were acyclic and thus had constant low endogenous estrogen levels. These older RS F exhibited reduced maximal constrictor responses to VP, which can be attributed to enhanced COX-1 derived dilator prostanoid production. VP-induced vasoconstriction in younger MA F (which exhibited estrous cycling) utilized both COX-1 and COX-2 derived constrictor prostanoids. Additionally, high concentration VP-stimulated PGI_2_ and TXA_2_ production were enhanced by endogenous estrogen and decreased with advancing age in F but not in M rat MCA.

Plasma estrogen levels measured in MA F rats in the present study were nearly double those of acyclic RS F rats. In previous studies, randomly cycling female Sprague–Dawley rats exhibited plasma estrogen levels significantly higher than cycling MA F rats in the present study. These higher values are reflective of surge as well as non-surge plasma estrogen levels [9], whereas the lower values in MA F rats in the present study are representative of uniformly non-surge levels in diestrus and metestrus phases. However, it should be noted that previous studies have failed to detect any significant changes in vascular function with the fluctuations in plasma estrogen during the estrous cycle [14]. Although endogenous estrogen clearly exerts significant effects on vascular function [9],[17], these effects do not appear to vary with the cyclic changes during the estrous cycle.

### 4.1 Effect of age on mechanisms of cerebrovascular function

Endothelial dysfunction increases in men after age 40 and in women after age 55 [18]. While the exact cause of the decline in endothelial function is unknown, aging is usually associated with a reduction in the ability to elicit endothelium-dependent vasodilation in both animals and in humans [19],[20]. This loss of function occurs via numerous mechanisms leading to attenuated nitric oxide-mediated dilation [19],[20]. In addition to the age-related changes in nitric oxide, an enhancement of vasoconstrictor prostaglandins further potentiates age-dependent endothelial dysfunction. With advancing age, both COX-1 and COX-2 expression are upregulated by oxidative stress, whereas PGI_2_ receptor (IP) expression declines [21]–[24]. There is also indirect evidence suggesting that untransformed PGH_2_ (which interacts with the TXA_2_ (TP) receptor) is also augmented with aging due to COX-1/COX-2 upregulation [25],[26]. Several studies have reported increases in TXA_2_ and TXS mRNA in aorta and mesenteric arteries with age [23],[27]. The results of the present study confirm and extend these findings regarding effects of age on vascular function through alterations in the balance of dilator to constrictor prostanoids, by decreasing production of and/or reactivity to PGI_2_ and augmenting production of constrictor prostanoids PGH_2_ and TXA_2_, and that these effects of age also occur in the cerebrovasculature.

### 4.2 Effects of sex and estrogen on mechanisms of cerebrovascular function

Women tend to exhibit higher levels of cerebral blood flow (CBF) than men when they are younger, but this difference becomes less significant later in life, around the onset of menopause [28]. Additionally, CBF varies throughout the menstrual cycle [29] and is altered during pregnancy [30]. Thus, chronic exposure to estrogen positively alters cerebrovascular function, at least in younger F. Interestingly, there appear to be striking sex-differences in the modulation of cerebrovascular arterial tone, with M arteries exhibiting greater myogenic tone in response to increasing pressure as compared to F [2]. Numerous studies have reported that estrogen enhances the production and/or the sensitivity of cerebral arteries to the vasodilatory factors nitric oxide and PGI_2_[2],[3],[5],[31],[32]. In the cerebrovasculature, estrogen appears to shift the prostaglandin balance towards greater production of vasodilator prostanoids [5], and estrogen upregulates both COX-1 and PGIS, resulting in enhanced PGI_2_ production [3],[5],[31], at least in young F rats. Interestingly, TXA_2_ production was also slightly, yet significantly, elevated in young F animals with estrogen-treatment, perhaps due to increased COX-1 levels [31],[33]. The age- and sex-dependent shifts in dilator and constrictor prostanoids observed in the present study reveal important new and novel age- and sex-dependent roles for both constrictor and dilator prostanoids in cerebrovascular function and clearly establish important roles for COX-2- and sex-dependent constrictor prostanoid production, which are likely due to the effects of endogenous estrogen to upregulate constrictor prostanoid function in F rats [8],[9],[17].

### 4.3 Divergent effects of age and estrogen on cerebrovascular function: beneficial vs. deleterious

Earlier findings in human epidemiological [34]–[36] and experimental animal studies [37]–[39] led to the dogmatic view that estrogen replacement therapy exerts beneficial effects on neurological and cardiovascular health and that it is protective against diseases such as dementia, coronary artery disease, hypertension, and stroke. An abundance of evidence from experimental animal studies has established that estrogen does exert beneficial or protective effects on the cerebrovasculature by reducing vascular reactivity and thereby increasing blood flow through nitric oxide- and vasodilator prostanoid-dependent mechanisms [2]–[5],[31],[40],[41], at least in younger animals. In contrast, more recent human epidemiological findings, such as the HERS [42], HERS II [43], and WHI studies [44], all suggest that, in older women, estrogen replacement therapy increases the incidences of neurological (dementia and stroke) and vascular (coronary artery disease, hypertension, and venous thrombosis) diseases. Thus, the role of estrogen in cardiovascular health and disease has become controversial. Indeed, recent studies of the systemic vasculature have clearly established that endogenous estrogen exerts deleterious effects on the F vasculature through upregulation of COX-2, TXS, and TP receptor expression, thereby enhancing production of, and responsiveness to, constrictor prostanoids in the F systemic vasculature [8],[9],[17]. Although numerous studies have investigated the beneficial effects of estrogen replacement in cerebral and systemic vascular beds of young animals [3],[5],[17], the present study is the first to examine the divergent effects of endogenous estrogen on cerebrovascular reactivity and prostanoid production in M and F rats at both middle and advanced ages. These findings suggest that aging and female sex both exert deleterious effects on cerebrovascular function through the modulation of constrictor and dilator prostanoid production, which appear to be synergistic in nature, and are likely due to the effects of endogenous estrogen in the F rat.

## 5 Conclusions

The results of this study provide important new and novel information on the effects of estrogen and advancing age on cerebrovascular function. Further understanding of the mechanisms by which estrogen exerts its beneficial vs. detrimental effects on the cerebrovasculature will perhaps lead to new age- and sex-specific therapeutic agents designed specifically to target the cerebrovasculature and other estrogen-responsive tissues.

## Abbreviations

COX-1: cyclooxygenase-1

COX-2: cyclooxygenase-2

CTL: control

eNOS: endothelial nitric oxide synthase

F: female

KHB: Krebs-Henseleit bicarbonate

M: male

MA: multigravid adult

MCA: middle cerebral artery

PGI_2_: prostacyclin

PSS: physiological salt solution

RS: reproductively senescent

TXA_2_: thromboxane

VP: vasopressin

## Competing interests

The authors declare that they have no competing interests.

## Authors’ contributions

RRD and JNS designed the project. RRD performed the experiments and analyzed the data. RRD and JNS interpreted the data and drafted and revised the article. Both authors read and approved the final manuscript.
